# Fibroblast growth factor modulates mast cell recruitment in a murine model of prostate cancer

**DOI:** 10.18632/oncotarget.19773

**Published:** 2017-08-01

**Authors:** Roberto Ronca, Roberto Tamma, Daniela Coltrini, Simona Ruggieri, Marco Presta, Domenico Ribatti

**Affiliations:** ^1^ Department of Molecular and Translational Medicine, University of Brescia, Brescia, Italy; ^2^ Department of Basic Medical Sciences, Neurosciences and Sensory Organs, University of Bari Medical School, Bari, Italy; ^3^ National Cancer Institute “Giovanni Paolo II”, Bari, Italy

**Keywords:** angiogenesis, fibroblast growth factor-2, long pentraxin-3, mast cell, prostate cancer

## Abstract

Mast cells are important modifiers of prostate tumor microenvironment. The fibroblast growth factor/fibroblast growth factor receptor (FGF/FGFR) system plays a non-redundant autocrine/paracrine role in the growth, vascularization and progression of prostate tumors. Accordingly, the FGF antagonist long pentraxin-3 (PTX3) and the PTX3-derived small molecule FGF-trap NSC12 have been shown to inhibit the growth and vascularization of different FGF-dependent tumor types, including prostate cancer. In this study, we show that recombinant FGF2 is able to cause mast cell recruitment *in vivo* in the Matrigel plug assay. Conversely, PTX3 overexpression in transgenic mice or treatment with the FGF inhibitor NSC12 result in a significant inhibition of the growth and vascularization of TRAMP-C2 tumor grafts, a murine model of prostate cancer, that were paralleled by a decrease of mast cell infiltrate into the lesion. These data confirm and extend previous observations about the capacity of mast cells to respond chemotactically to FGF2 stimulation and provide evidence about a relationship among mast cell recruitment, angiogenesis, and tumor growth in human prostate adenocarcinoma.

## INTRODUCTION

A tight cross-talk exists between inflammation and cancer, tumor-infiltrating inflammatory cells producing various cytokines that regulate the inflammatory response in the tumor-bearing host. Among inflammatory cells identified as important modifiers of tumor microenvironment, mast cells appear to play a crucial role [1]. Indeed, mast cells express a variety of growth factors/angiogenic mediators and their accumulation correlates with increased neovascularization, tumor aggressiveness and poor prognosis in different solid and hematologic tumors [1].

Prostate tumors are a leading cause of death in the male population of Western countries [2]. Androgen receptor signalling plays a central role in prostate cancer, androgen deprivation therapy representing the first line of treatment for the advanced disease. However, tumors will eventually progress to an androgen-independent stage with poor prognosis. A correlation has been established between mast cell density and microvessel density in human prostate adenocarcinoma [3]. Tryptase-positive mast cells are present in both intratumoral and peritumoral areas of prostate adenocarcinoma with an overlapping mean density [4]. In addition, mast cell number in the intratumoral and peritumoral regions of prostate adenocarcinoma is significantly higher as compared to benign lesions [5]. Moreover, mast cells promote well-differentiated adenocarcinoma growth in human patients and in the autochthonous transgenic adenocarcinoma of the mouse prostate (TRAMP) model [6].

The members of the fibroblast growth factor (FGF) gene family [7] exert their activity by binding to tyrosine kinase FGF receptors (FGFRs) [8]. The FGF/FGFR system plays a non-redundant autocrine/paracrine role in the growth, vascularization and progression of prostate tumors towards an hormone-refractory state as the consequence of the deregulation of FGFR signalling [9–14]. Indeed, prostate tumors express various FGF family members, including FGF2 and FGF8b, with paracrine/autocrine functions on cancer epithelial/stromal cells [11, 15, 16]. In particular, the FGF/FGFR system plays a prominent role during the growth of tumors obtained after grafting of androgen-dependent prostate TRAMP-C2 cells in syngeneic mice [17–19]. Thus, TRAMP-C2 cells, originated from TRAMP mice [20], represent a model system suitable for the study of the effects of FGF/FGFR antagonists in the treatment of prostate cancer [21].

Long pentraxin-3 (PTX3) is a member of the pentraxin family produced locally in response to inflammatory signals [22]. PTX3 shares the *C*-terminal domain with short pentraxins and possesses a unique *N*-terminal domain [22]. When assessed for the capacity to interact with a variety of growth factors/cytokines, PTX3 was found to bind FGF2 *via* its *N*-terminal extension [23, 24], thus inhibiting FGF2-dependent endothelial cell proliferation *in vitro* and angiogenesis *in vivo* [23–26]. In addition, PTX3 was found to inhibit the growth of various FGF-dependent tumor types (reviewed in [27]). On this basis, pharmacophore modeling of the interaction of a minimal PTX3-derived FGF-binding pentapeptide with FGF2 has been used for the identification of the first small molecule chemical (NSC12) which acts as an extracellular FGF trap with significant implications in cancer therapy [27].

In particular, previous observations had shown that PTX3 overexpression suppresses the angiogenic and tumorigenic potential of TRAMP-C2 cells by inhibiting the FGF-dependent autocrine/paracrine loop of stimulation driven by dihydrotestosterone [28]. Accordingly, injection of TRAMP-C2 cells in transgenic male mice overexpressing PTX3 under the control of the endothelial *Tie2*-promoter results in a significant inhibition of the growth and vascularization of the tumor graft [27]. Similar results were obtained when TRAMP-C2 cells were grafted in wild-type mice treated with the PTX3-derived small molecule FGF-trap [27].

Mast cells express FGFRs [29] and respond chemotactically to FGF2 stimulation [30]. Thus, the aim of this study was to investigate the capacity of FGF2 to induce an angiogenic response and mast cell recruitment *in vivo* in a murine Matrigel plug assay. Moreover, we have analyzed the impact of the inhibition of the FGF/FGFR system by genetic and pharmacologic approaches on mast cell recruitment in prostate cancer. In a first set of experiments, FGF-dependent TRAMP-C2 cells were injected s.c. in syngeneic wild-type and in PTX3-overexpressing transgenic TgN(Tie2-hPTX3) male mice. In a second set of experiments, TRAMP-C2 cells were grafted s.c. in wild type mice that were treated i.p. with the pan-FGF trap NSC12 inhibitor or the control compound NSC21. In both experimental conditions, immunohistochemical analysis was performed on harvested tumors to evaluate mast cell density in peripheral and central areas of the grafts and to correlate these values with the neovascular response in the same areas.

## RESULTS

### FGF2 recruits mast cells in the *in vivo* Matrigel plug assay

**Figure 1:**
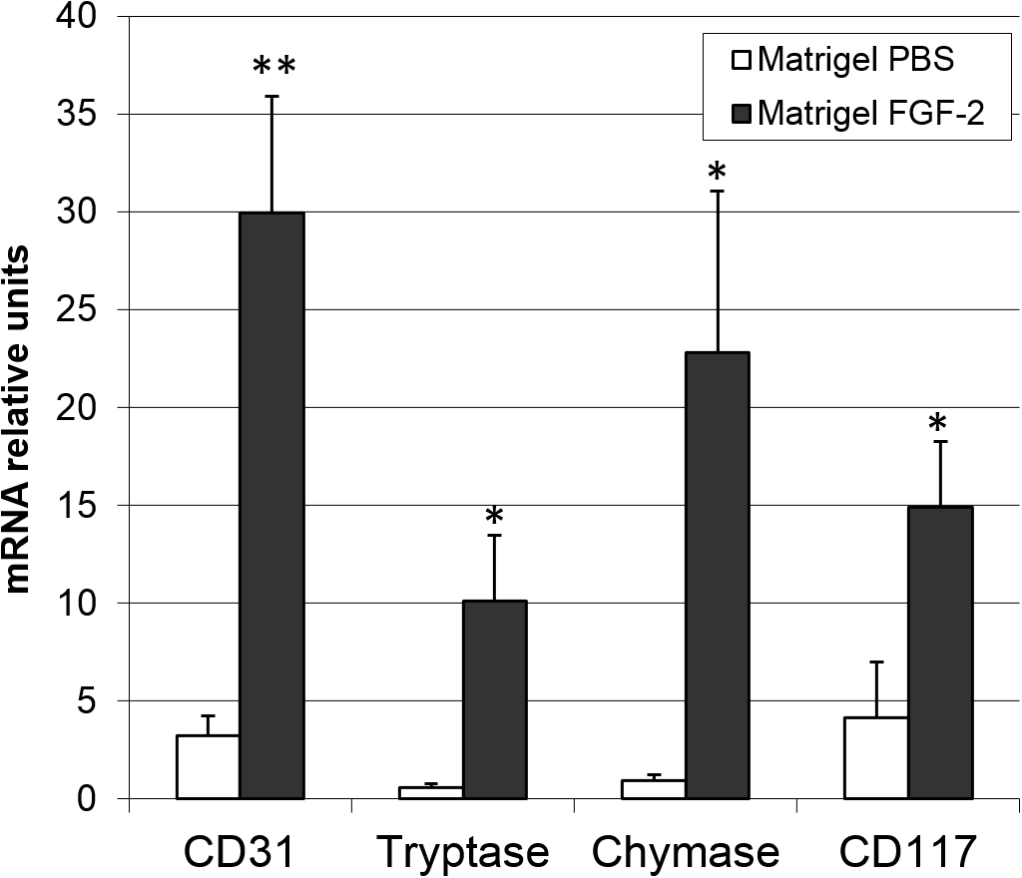
FGF2 induces an angiogenic response and mast cell recruitment *in vivo* Matrigel plugs (n = 4–8) containing PBS or 4.0 pmoles of FGF2 were implanted in the flank of female C57BL/6 mice. After 7 days, plugs were harvested and the levels of expression of lineage specific endothelial marker CD31 and mast cell tryptase, chymase and CD117 markers were assessed by qPCR analysis. Data are means ± SEM. *p < 0.05; **p < 0.01 versus PBS; Student’s *t* test.

The capacity of FGF2 to induce an angiogenic response and mast cell recruitment was investigated *in vivo* by quantitative PCR analysis applied to a murine Matrigel plug assay [31]. As shown in Figure 1, FGF2-treated plugs exert a remarkable neovascular and mast cell chemoattractant response, as showed by the significant upregulation of the expression of lineage specific endothelial CD31 and mast cell tryptase, chymase and CD117 transcripts in respect to control plugs. These data extend previous observations about the capacity of FGF2 to induce a chemotactic response on mast cells *in vitro* [30].

### Impact of anti-FGF approaches on the growth of prostate TRAMP-C2 tumor grafts

**Figure 2:**
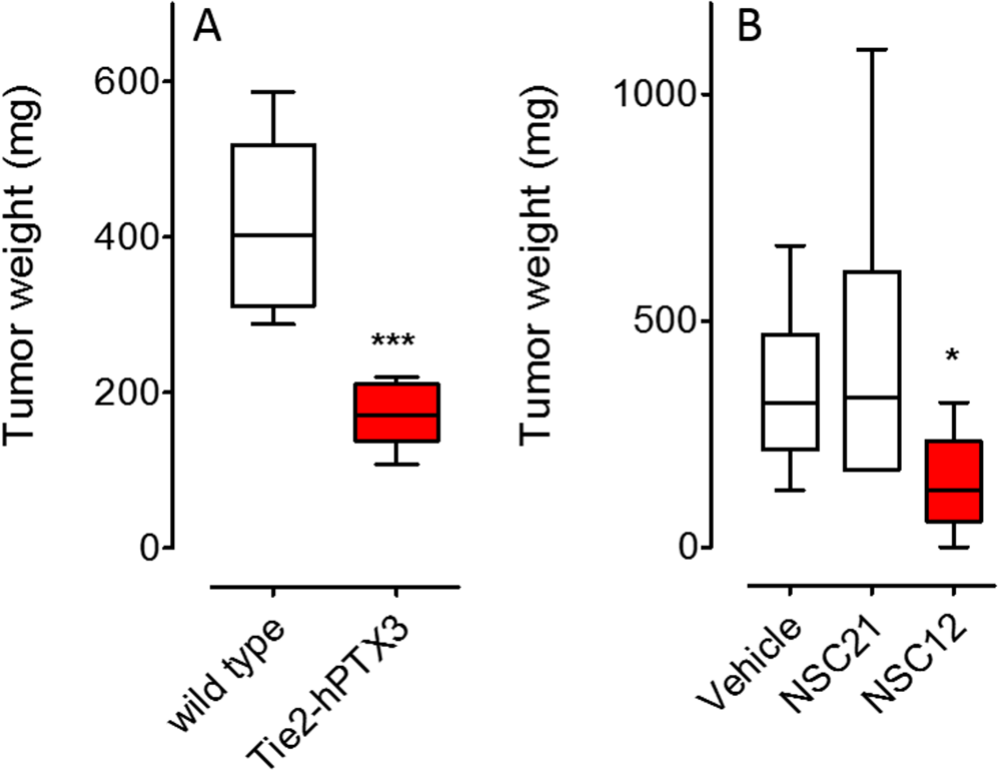
Inhibition of prostate TRAMP-C2 tumor grafts by FGF inhibitors **(A)** FGF-dependent TRAMP-C2 cells were injected s.c. in syngeneic wild-type and in PTX3-overexpressing transgenic TgN(Tie2-hPTX3) male mice that were sacrificed 37 days after grafting (n = 6). **(B)** TRAMP-C2 cells were grafted s.c. in wild type mice that were treated i.p., twice a week, with 7.5 mg/Kg of the pan-FGF trap NSC12 or the control compound NSC21 starting from day 30 (n = 6-8) and sacrificed at day 49. Tumors were harvested, weighted and processed for IHC analysis. Data are means ± SEM. *P < 0.05; ***P < 0.001; Student’s *t* test.

Genetic and pharmacologic anti-FGF approaches were used to investigate the impact of the inhibition of the FGF/FGFR system on mast cell recruitment in prostate cancer. In a first set of experiments, FGF-dependent TRAMP-C2 cells were injected s.c. in syngeneic wild-type and PTX3-overexpressing transgenic TgN(Tie2-hPTX3) male mice. In a second set of experiments, TRAMP-C2 cells were grafted s.c. in wild type mice that were treated i.p. with the pan-FGF trap inhibitor NSC12 (Supplementary Figure 1) or the control compound NSC21 [27]. In agreement with previous observations [27], both anti-FGF approaches resulted in a significant reduction of tumor weight at sacrifice (Figure 2). On this basis, harvested grafts were analyzed for vascularization and mast cell infiltration by immunohistochemistry.

### PTX3 overexpression inhibits neovascularization and mast cell recruitment in prostate TRAMP-C2 tumor grafts

**Figure 3:**
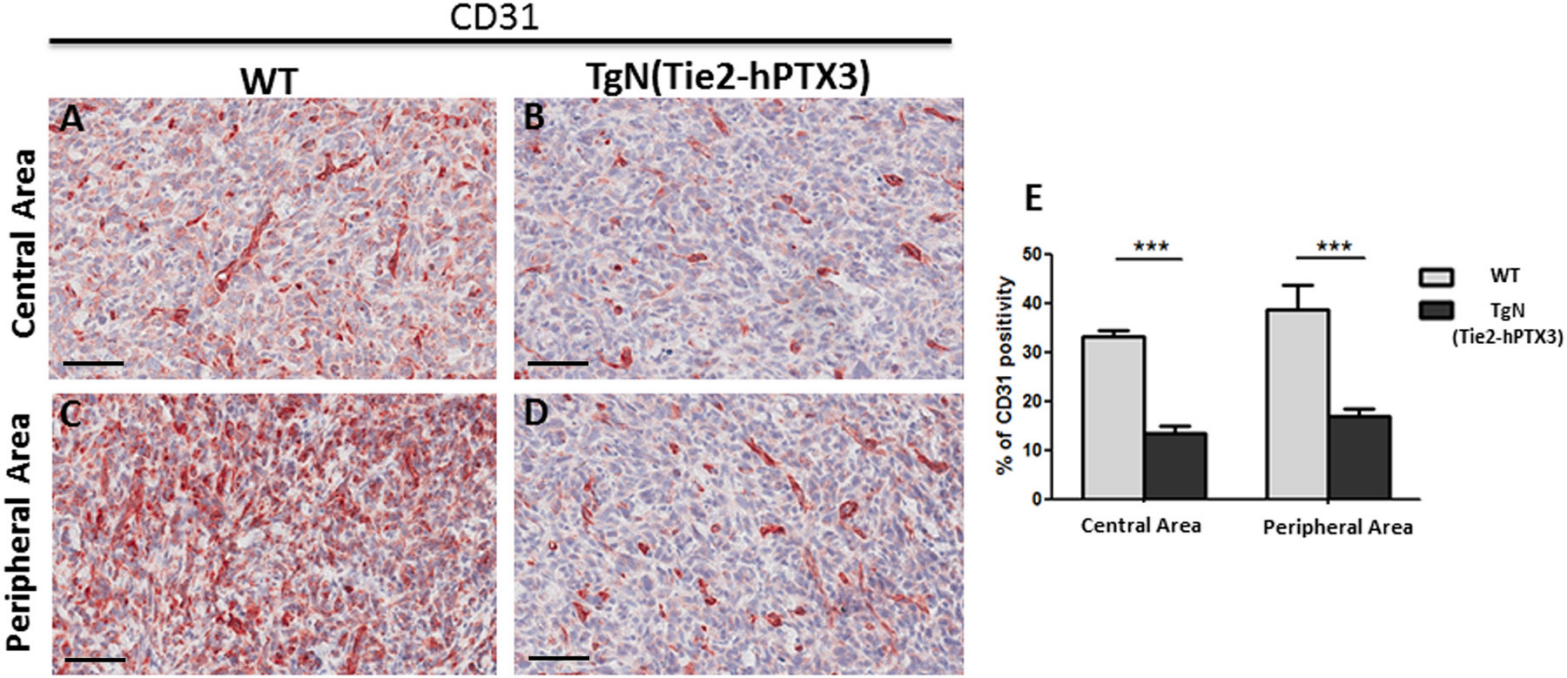
Immunohistochemical **(A-D)** and morphometric analysis **(E)** of CD31 immunoreactivity in central (A-B) and peripheral (C-D) areas of TRAMP-C2 grafts harvested from wild type (WT) (A-C) and TgN(Tie2-hPTX3) (C-D) mice. The percentage of CD31-positive endothelial cells (E) decreases significantly in both central (B) and peripheral (D) areas of TgN(Tie2-hPTX3) samples as compared to WT samples (***p< 0.001). Scale bars: A-D, 60 μm. Data are reported as means ± SEM. Bonferroni post-test was used to compare all treatment groups after one-way ANOVA.

**Figure 4:**
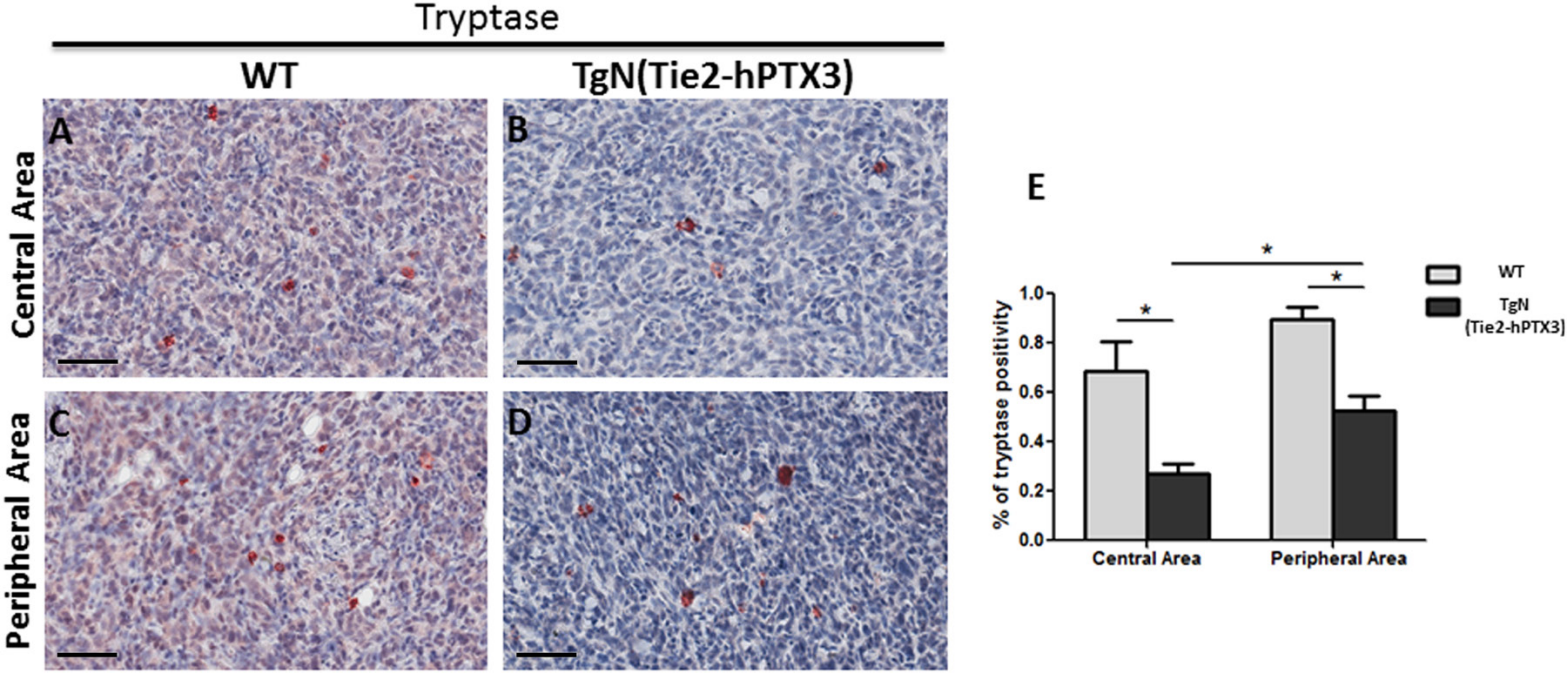
Immunohistochemical **(A-D)** and morphometric analysis **(E)** of tryptase immunoreactivity in central (A-B) and peripheral (C-D) areas of TRAMP-C2 grafts harvested from wild type (WT) (A-C) and TgN(Tie2-hPTX3) (C-D) mice. The percentage of tryptase-positive mast cells (E) decreases significantly in both central (B) and peripheral (D) areas of TgN(Tie2-hPTX3) samples as compared to WT samples (*p< 0.05). Scale bars: A-D, 60 μm. Data are reported as means ± SEM. Bonferroni post-test was used to compare all treatment groups after one-way ANOVA.

Immunohistochemical analysis was performed on sections of TRAMP-C2 tumor lesions originated in wild type (WT) and TgN(Tie2-hPTX3) mice in order to identify CD31-positive endothelial neovessels (Figure 3A-3D) and the tryptase-positive mast cell infiltrate (Figure 4A-4D) in the central and peripheral areas of tumor grafts. As shown in Figure 3, the density of CD31-positive cells was decreased in both central (13.56% ± SE 1.59%) and peripheral (17.50% ± SE 1.50%) areas of tumors harvested from TgN(Tie2-hPTX3) mice as compared to grafts obtained in WT animals (33.18% ± SE 1.33%, center; 38.79 ± SE 4.9%, periphery). Accordingly, tryptase-positive mast cell infiltrate was less abundant in both central and peripheral areas of TRAMP-C2 tumor grafts generated in transgenic mice (0.27% ± SE 0.04% and 0.52% ± SE 0.08%, respectively) as compared to that measured in WT animals (0.68% ± SE 0.12% and 0.89% ± SE 0.05%, respectively) (Figure 4). Notably, mast cell infiltrate at the periphery of TRAMP-C2 tumor lesions grown in TgN(Tie2-hPTX3) mice appears to be more abundant than in the central areas of the lesion (*p< 0.05).

### A small molecule FGF trap inhibits neovascularization and mast cell recruitment in prostate TRAMP-C2 tumor grafts

**Figure 5:**
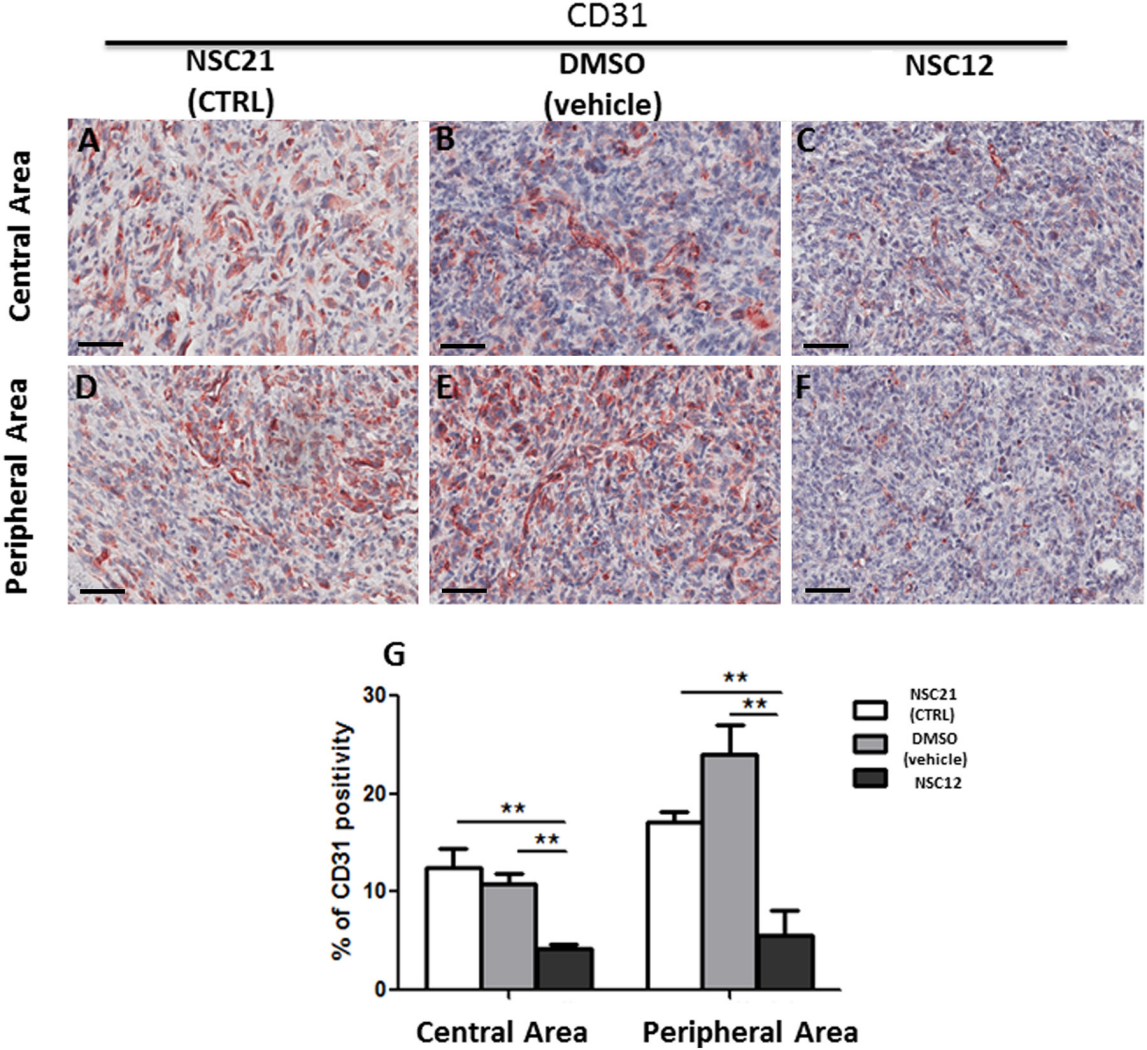
Immunohistochemical **(A-F)** and morphometric analysis **(G)** of CD31 immunoreactivity in central (A-C) and peripheral (D-F) areas of TRAMP-C2 grafts harvested from wild-type mice treated i.p. with NSC21 (A-D), DMSO (B, E) or NSC12 (C-F). The percentage of CD31-positive endothelial cells (G) decreases significantly in both central (C) and peripheral (F) areas of NSC12-treated tumors as compared to DMSO- or NSC21-treated lesions (**p< 0.01) (A, B, D, E). Scale bars: A-D, 60 μm. Data are reported as means ± SEM. Bonferroni post-test was used to compare all treatment groups after one-way ANOVA.

**Figure 6:**
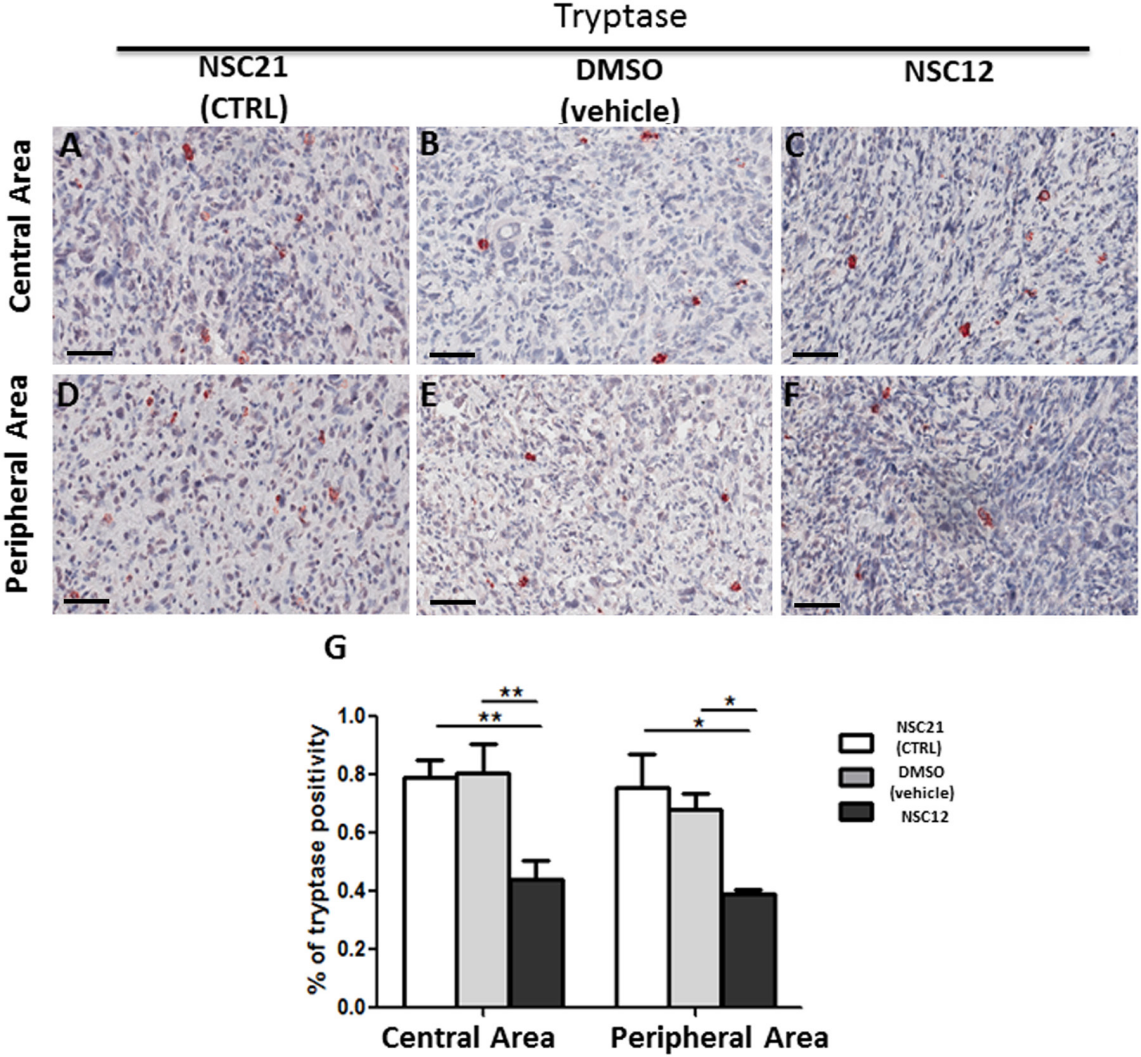
Immunohistochemical **(A-F)** and morphometric analysis **(G)** of tryptase immunoreactivity in central (A-C) and peripheral (D-F) areas of TRAMP-C2 grafts harvested from wild-type mice treated i.p. with NSC21 (A-D), DMSO (B, E) or NSC12 (C-F). The percentage of tryptase-positive mast cells (G) decreases significantly in both central (**p< 0.01) and peripheral (*p< 0.05) areas of NSC12-treated tumors (C, F) as compared to DMSO- or NSC21-treated lesions (A, B, D, E) (A-C, D-F). Scale bars: A-D, 60 μm. Data are reported as means ± SEM. Bonferroni post-test was used to compare all treatment groups after one-way ANOVA.

Similar to PTX3 overexpression, administration of the PTX3-derived small molecule FGF trap NSC12 results in a significant inhibition of neovessel density and mast cell recruitment in TRAMP-C2 lesions generated by s.c. injection of tumor cells in syngeneic wild-type mice when compared to grafts grown in animals treated with vehicle (DMSO) or the control compound NSC21 (Figures 5 and 6). Indeed, the density of CD31-positive endothelial cells was decreased in both central (4.26% ± SE 0.45%) and peripheral (5.56% ± SE 2.55%) areas of NSC12-treated samples as compared to NSC21-treated (12.54% ± SE 1.89%; 17.15± SE 1.02%) or DMSO-treated (10.89% ± SE 0.92%; 24.09 ± SE 2.86%) specimens (Figure 5). Similarly, tryptase-positive mast cell infiltrate was decreased in NSC12 samples in both central (0.44% ± SE 0.06%) and peripheral (0.39% ± SE 0.01%) areas as compared to NSC21 (0.79% ± SE 0.05%; 0.75± SE 0.11%) and DMSO (0.8% ± SE 0.09%; 0.68 ± SE 0.01%) specimens (Figure 6).

## DISCUSSION

The significant upregulation of the expression of the mast cell markers tryptase, chymase and CD117 in FGF2-treated Matrigel plugs in respect to controls demonstrates that recombinant human FGF2 is able to induce a significant recruitment of mast cells *in vivo* when delivered s.c. in mice via a Matrigel plug. In addition, in keeping with the potent pro-angiogenic activity of this growth factor, mast cell recruitment was paralleled by a significant upregulation of the endothelial marker CD31 in FGF2 plugs when compared to controls. Conversely, our data indicate that genetic or pharmacologic inhibition of the FGF/FGFR system in a murine model of prostate cancer results in a significant inhibition of tumor growth and neovascularization that are constantly associated to a reduction of mast cell density in both the peripheral and central areas of the tumor. These data confirm and extend previous observations about FGFR expression in mast cells [29] and the capacity of these cells to respond chemotactically to FGF2 stimulation *in vitro* [30]. In addition, they provide indirect evidence about a relationship among mast cell recruitment, angiogenesis, and tumor growth in human prostate adenocarcinoma. Although mast cell recruitment in the tumor tissues was reduced by FGF blockade, the decrease in vascularization appears to be more pronounced when compared to the reduction in the mast cell infiltrate. This may represent the consequence of the multi-targeting effects of anti-FGF approaches in the control of tumor angiogenesis that may result in a direct inhibition of endothelial cells as well as of tumor and infiltrating stromal cells, including mast cells.

As observed for tumor-associated macrophages (TAMs), mast cells may exert both pro- and anti-tumor effects [32]. Indeed, mast cells induce immunosuppression by releasing tumor necrosis factor alpha (TNF-α) and interleukin10 (IL-10), involved in promoting the immune tolerance mediated by regulatory T cells [33, 34]. In addition, mast cells may promote inflammation, inhibition of tumor cell growth, and tumor cell apoptosis by releasing interleukin (IL)-1, IL-4, IL-6, IL-8, monocyte chemotactic protein-3 and-4 (MCP-3 and MCP-4), transforming growth factor beta (TGF-β), and chymase [1].

In prostate cancer, Fleischmann et al. [35] found a strong association among high mast cell counts and Gleason score, early tumor stage, and low risk for recurrence. At variance, Nonomura et al. [36] reported that a decreased number of mast cells correlates with a better prognosis in prostate cancer. Notably, Johansson et al. [37] demonstrated that prostate peritumoral mast cells stimulate angiogenesis and tumor growth by secreting FGF2 whereas intratumoral mast cells correlate negatively with the presence of metastases, tumor stage, Gleason score, angiogenesis, and tumor cell proliferation, being associated to a good prognosis. Mast cells synthesize and release potent angiogenic cytokines, including vascular endothelial growth factor (VEGF), FGF2, the serine proteases tryptase and chymase, IL-8, TGF-β, TNF-α and nerve growth factor (NGF) [40]. Mast cell-secreted molecules facilitate tumor vascularization not only by a direct angiogenic effect but also by stimulating other inflammatory cells of the tumor microenvironment to release angiogenic mediators and cytokines as well as extracellular matrix-degrading proteases. Indeed, an increased number of mast cells have been demonstrated to be associated with angiogenesis in a variety vascular, haematological and solid tumors [40].

Mast cells have been indicated as a novel prognostic marker and a target for therapy associated with castration in prostate cancer [37]. In patients, mast cells are recruited to the tumoral compartment during the formation of castrate-resistant prostate tumors. In a rat orthotopic prostate tumor model, mast cells infiltrate tumors that relapse after an initial response to castration treatment. This occurs in association with increased angiogenesis and FGF2 upregulation [38, 39].

Mast cells might act as a new target for the adjuvant treatment of tumors through the selective inhibition of angiogenesis, tissue remodeling and tumor promoting molecules, allowing the secretion of cytotoxic cytokines and preventing mast cell mediated immune-suppression. Pre-clinical studies using anti-c-kit antibodies [40], anti-TNF-α antibodies [41], or the mast cells stabilizer disodium cromoglycate (cromolyn) [42] have demonstrated promising results in mouse models. Masatinib is a tyrosine kinase inhibitor that targets c-kit receptors and is clinically developed and approved for treatment of recurrent or unresectable grade III dog mast cell tumors and is the first approved anticancer drug in veterinary medicine [43]. Masatinib has been translated to human clinical trials for its evaluation in gastro intestinal stromal tumor (GIST), mastocytosis and pancreatic cancer [44, 45].

Here, in agreement with previous findings [27], we show that PTX3 overexpression or treatment with the PTX3-derived small molecule FGF trap NSC12 result in a significant inhibition of the growth of TRAMP-C2 tumor grafts, a murine model of prostate cancer [20]. This was paralleled by a decrease of mast cell infiltrate into the lesion. This may represent the result of a multi-target effect of these anti-FGF approaches on different tumor compartments that may cause the inhibition of FGF-mediated mast cell recruitment into the tumor and the inhibition of the mitogenic and angiogenic activity exerted by FGF molecules produced by the tumor parenchyma and stroma, including recruited mast cells. Even though further studies will be required to elucidate this complex interplay, our observations indicate that small FGF trap molecules may represent the basis for novel approaches in the therapy of prostate cancer [46]. In conclusion, this work provides further insights about the role of the FGF/FGFR system and mast cells in the control of prostate tumor growth and angiogenesis, demonstrating for the first time in an *in vivo* prostate tumor model that mast cell recruitment at the tumor site and tumor neovascularization are two strictly FGF-dependent correlated events.

## MATERIALS AND METHODS

### Reagents and cell cultures

Compound NSC12 was synthesized by M. Mor (University of Parma, Italy) as described [47] and control compound NSC21 was provided by Drug Synthesis and Chemistry Branch, Developmental Therapeutics Program, Division of Cancer Treatment and Diagnosis, National Cancer Institute (USA). Human recombinant FGF2 was from Tecnogen (Caserta, Italy). Murine prostate adenocarcinoma TRAMP-C2 cells were obtained from ATCC repository and maintained in DMEM supplemented with 10% heat inactivated FBS, 10 mM HEPES buffer, 0.5 mM 2-mercaptoethanol, 2.0 mM glutamine, 5 mg/L bovine insulin (Sigma-Aldrich) and 10 nM DHT as described [28].

### Matrigel plug angiogenesis assay

Six-week-old female C57BL/6 mice were injected subcutaneously (s.c.) with 400 μl of Matrigel (Trevigen, Gaithersburg, MD) containing PBS or 4.0 pmoles of FGF2. After 7 days, pellets were processed for total RNA extraction after adding to each plug a fixed amount of human cells as an internal standard [33]. Then, samples were analyzed for the expression of the indicated genes by real time quantitative PCR (qPCR) and data were normalized for human *GAPDH* expression levels as described [33]. To this purpose, total RNA was extracted from Matrigel plugs using TRIzol Reagent according to manufacturer’s instructions (Invitrogen). Contaminating DNA was digested using DNAse (Promega), 2.0 μg of total RNA were retro-transcribed with MMLV reverse transcriptase (Invitrogen) using random hexaprimers. Quantitative PCR was performed with a ViiATM 7 Real-Time PCR Detection System (Applied Biosystems) using iQTM SYBR Green Supermix (Biorad) according to manufacturer’s instructions. In each experiment, an arbitrary value equal to 1.0 was assigned to the levels of expression of the gene(s) measured in one PBS Matrigel sample that was used as reference.

The following primers were used: *murine CD31*, 5’-CGGTTATGATGATGTTTCTGGA (forward) and 5’-AAGGGAGGACACTTCCACTTCT (reverse); *murine Tryptase beta 2*, 5’- CCTCTCCCACCTCCTTATCC (forward) and 5’-CCCTTCACTTTGCAGACCAG (reverse); *murine Chymase 1*, 5’ GGCAGAACAAACGTGAATGA (forward) and 5’-CTATCCCAGCACACAGCAGA (reverse); *murine CD117*, 5’-GTGAACCAACTTCGCCTGAC (forward) and 5’- GAATCCCTCTGCCACACACT (reverse); *human GAPDH*, 5’- GAAGGTCGGAGTCAACGGATT (forward) and 5’- TGACGGTGCCATGGAATTTG (reverse).

### *In vivo* tumor models

Wild-type C57BL/6 mice were purchased from Envigo (Correzzana, Italy). Transgenic TgN(Tie2-hPTX3) mice overexpressing hPTX3 under the control of the endothelial specific *Tie2* promoter were generated as described [27]. In a first set of experiments, nine-week-old wild-type and TgN(Tie2-hPTX3) male mice were injected s.c. into the dorsolateral flank with 5x10^6^ TRAMP-C2 cells in 200 μl total volume of PBS and sacrificed at day 37. In a second set of experiments, 5x10^6^ TRAMP-C2 cells were injected s.c. into the dorsolateral flank of wild-type mice. After 30 days, vehicle (DMSO), NSC12 or NSC21 (both at 7.5 mg/kg) were injected i.p., twice a week, in 100 μl final volume. At the end of the experimental procedure (49 days after tumor cell grafting), tumors were harvested, weighted and paraffin embedded for immunohistochemical analysis of paraffin-embedded tumor sections.

### CD31 and tryptase immunohistochemistry

Histological sections (4 μm thickness) were collected on poly-L-lysine-coated slides (Sigma Chemical, St Louis, MO, USA) and deparaffinized. Sections were rehydrated in a xylene-graded alcohol scale and then rinsed for 10 minutes in 0.1 M PBS. Sections were pre-treated with sodium citrate pH 6.1 (Dako Corporation, Milan, Italy) in Dako PT Link for antigen retrieval solution for 30 minutes at 98°C and then incubated with rabbit polyclonal anti-CD31 antibody (ab28364, Abcam, Cambridge, UK) or mouse monoclonal anti-tryptase antibody (NB-100-64820, Novus Biologicals, Littleton, CO, USA) diluted 1:60 and 1:1000, respectively. Then, sections were counterstained with Mayer hematoxylin and mounted in synthetic medium. Specific preimmune serum (Dako), replacing the primary antibodies, served as negative control. Sections from each experimental group (n = 10; 10 cases per group) were scanned using the whole-slide morphometric analysis scanning platform Aperio Scanscope CS (Leica Biosystems, Nussloch, Germany). All the slides were scanned at the maximum available magnification (40×) and stored as digital high resolution images on the workstation associated with the instrument. Digital slides were inspected with Aperio ImageScope v.11 software (Leica Biosystems, Nussloch, Germany) at 20× magnification and 10 fields with an equal area were selected for the analysis at 40× magnification. The protein expression was assessed with the Positive Pixel Count algorithm embedded in the Aperio ImageScope software and reported as positivity percentage, defined as the number of positively stained pixels on the total pixels in the image.

### Statistical analysis

Data related to the experimental groups, WT and TgN(Tie2-hPTX3), NSC12, NSC21 and vehicle samples are reported as means ± SEM. Bonferroni post-test was used to compare all treatment groups after one-way ANOVA. The Graph Pad Prism 5.0 statistical package (GraphPad Software, San Diego, CA, USA) was used for analyses and the limit for statistical significance was set at P<0.05.

## SUPPLEMENTARY MATERIALS FIGURE



## Abbreviations

FGF: fibroblast growth factor
FGFR: fibroblast growth factor receptor
PTX3: pentaxin3
TRAMP-C2: transgenic adenocarcinoma of the mouse prostate –C2 cell line
Tie2: tyrosine kinase with Ig and EGF (epidermal growth factor) homology domains-2
s.c.: subcutaneous
i.p.: intraperitoneal
CD31/117: cluster of differentiation - 31, -117
TAMs: tumor-associated macrophages
MCP3/4: monocyte chemotactic protein-3, -4
TGF-β: transforming growth factor beta
TNF-α: tumor necrosis factor- α
VEGF: endothelial growth factor
NGF: nerve growth factor
Ckit: Proto-Oncogene Proteins c-kit
