# Supplementary material for: Fibroblast growth factor modulates mast cell recruitment in a murine model of prostate cancer
Source: Oncotarget. 2017 Aug 1;8(47):82583–92. doi: 10.18632/oncotarget.19773 (PMC5669912; doi:10.18632/oncotarget.19773)
Supplement: Supplementary file 1 [file oncotarget-08-82583-s001.pdf]

Fibroblast growth factor modulates mast cell recruitment in a murine model of prostate cancer

SUPPLEMENTARY MATERIALS

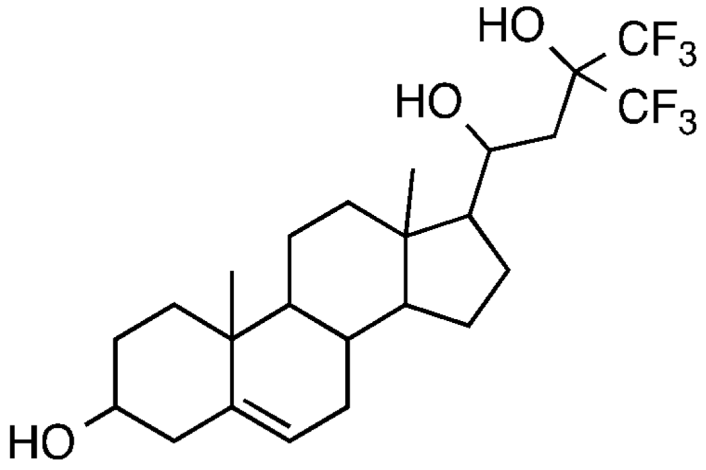

Supplementary Figure 1: Chemical structure of the FGF trap NSC12.
